# Revealing the sedative-hypnotic effect of the extracts of herb pair Semen Ziziphi spinosae and Radix Polygalae and related mechanisms through experiments and metabolomics approach

**DOI:** 10.1186/s12906-020-03000-8

**Published:** 2020-07-02

**Authors:** Hong Luo, Sheng-jie Sun, Yan Wang, Ying-li Wang

**Affiliations:** School of Chinese Materia Medica, Shanxi University of Chinese Medicine, Daxue Street, No.121, Jinzhong, Shanxi China

**Keywords:** GC-TOF-MS, Herbal pair, Sedative-hypnotic effect, Serum metabolomics approach, Semen Ziziphi spinosae and Radix Polygalae

## Abstract

**Background:**

Semen Ziziphi spinosae and Radix Polygalae, two herbs commonly used together in Traditional Chinese Medicine for the treatment of insomnia and anxiety. The study aims to study the sedative-hypnotic effect of the active components of the herbal pair, the possible mechanisms of such effect, and related metabolic pathways in vivo.

**Methods:**

The sedative and hypnotic effect of the active components (EI30) of the herbal pair was studied by recording influence on the proportion of sleeping within 30 min, sleep latency and sleep length of pentobarbital sodium-induced sleeping on mice. Possible mechanisms of the sedative-hypnotic effect of the active components were investigated by measuring the content of neurotransmitters in the total protein of mice brain tissue. The main chemical compounds of the herbal pair were identified by Liquid Chromatography-Mass Spectrometry (LC-MS). Serum samples of mice were studied, and related differential metabolites between the normal group and model group, and between model group and treatment group were identified by Gas Chromatography Time-Of-Flight Mass Spectrometry (GC-TOF-MS), Principal Components Analysis (PCA), and Orthogonal Projections to Latent Structures Discriminant Analysis (OPLS-DA).

**Results:**

Compared with the control group, high dose EI30 group and the Clonazepam group were with significantly higher proportions of sleep within 30 min (*P* = 0.027 and 0.005 respectively). Compared with the control group, all of the high, medium and low dose of EI30 groups were with significantly shorter sleep latency (*P* < 0.01) and prolonged sleeping time (*P* < 0.01). The herbal pair has good sedative-hypnotic effects, although it is weaker than the effect of Clonazepam. The sedative-hypnotic effect of EI30 is possibly related to the adjustment of neurotransmitters 5-hydroxytryptamine (5-HT), norepinephrine (NE), and dopamine (DA) in the total protein of mice brain tissue. There are five metabolic pathways in vivo most related to the sedative-hypnotic effect of EI30, and they are biosynthesis of valine, leucine, and isoleucine, metabolism of glyceride, metabolism of alanine, aspartic acid and glutamic acid, metabolism of phenylalanine, and metabolism of cysteine and methionine.

**Conclusions:**

This study reveals the mechanisms of sedative and hypnotic effects of herbal pair Semen Ziziphi spinosae and Radix Polygalae by using metabolomics methods. This study provides a basis for further development and utilization of this herbal pair.

## Background

Semen Ziziphi spinosae, the seed of *Ziziphus jujuba* Mill. var. *spinosa* (Bunge) Hu ex H. F. Chow, mainly contains substances such as flavonoids, saponins, alkaloids, volatile oils, amino acids [1, 2]. Semen Ziziphi spinosae has been proven by previous studies to have medicinal functions of sedative-hypnotic effect, anti-anxiety effect [3], anti-depression effect [4], hypoglycemic action [5], anti-dementia effect [6], and effect of strengthening the immune system [7], etc. The total flavonoids and total saponins in Semen Ziziphi spinosae are the principal effective components for sedative and hypnotic effects [8, 9].

One of the main effective compounds for the sedative and hypnotic effects of Semen Ziziphi spinosae is spinosin, which is a flavonoid glycoside monomer [10]. Spinosin can induce sleep in mice [11], prolong the length of pentobarbital induced sleep and reduce sleep latency in rats [12]. A previous animal experiment has shown that spinosin has anxiolytic-like effects in mice. High doses of spinosin (5 mg/kg/day) on mice could significantly increase the number of times entering into the open arms of the elevated plus-maze, the proportion of time spent on the open arms, the number of transitions between light and dark boxes, and time spent in the light compartment [13].

Jujuboside A found in Semen Ziziphi spinosae had been proven by a previous animal experiment to have sedative effects. A study showed that jujuboside A could reduce the activity of drosophilae melanogaster during the day and prolong their sleep time [14].

The alkaloids in Semen Ziziphi spinosae also showed obvious sedative effects, according to a previous study conducted on mice. The alkaloids in Semen Ziziphi spinosae can significantly inhibit spontaneous activities in mice, shorten the latency of sleep induced by a suprathreshold dose pentobarbital sodium, and prolong the sleep duration [15].

Radix Polygalae, dry roots of *Polygala tenuifolia* Willd or *Polygala sibirica* L. mainly contain substances such as saponins, sugar esters, volatile oils, organic acids, and small amounts of alkaloids and flavonol glycosides [16–19]. Radix Polygalae is reported to have effects of sedation and hypnosis, memory-enhancing [20], anti-dementia [21], anti-aging [22], and anti-inflammatory [23].

Senegenin is the active component for the sedative and hypnotic effects of Radix Polygalae [24]. Tenuifolin, a kind of senegenin*,* has been proven to have sedative effects. In a study, mice were intragastrically injected with tenuifolin at doses of 20, 40, and 80 mg/kg, and the results showed that 40 mg/kg and 80 mg/kg doses groups were with significantly increased the non-rem sleep time, rem sleep time, and total sleep time [25].

Other substances in Radix Polygalae had been proven to have sedative effects as well. The sedative-hypnotic effects of 3,4,5-Trimethoxycinnamic acid (TMAC), a compound derived from Radix Polygalae, were proven to act via increasing the chloridion influx and activating glutamic acid decarboxylase and γ-subunit of GABA_A_ receptors in the cerebellar granule cells [26].

Studies have shown that 3,6′-disinapoyl sucrose derived from Radix Polygalae also has sedative effects. It has a definite antidepressant effect and can improve the behavioral indexes of depression mice. Its effects may act via participating in the regulation of apoptosis genes, inhibiting apoptosis, and promoting nerve cell regeneration [27]. Another way for it to promote nerve regeneration and protection may be to participate in the regulation of cellular oxidative stress by improving the body’s antioxidant capacity and free radical scavenging capacity [27].

In traditional Chinese medicine, Semen Ziziphi spinosae and Radix Polygalae are commonly used as a herbal pair. A herbal pair refers to two herbs used together in clinical practice. They can act synergistically to enhance herb effects or relieve toxicity [28]. The results of preliminary research conducted by authors of this study showed that water extraction liquid of both Radix Polygalae and Semen Ziziphi spinosae could improve the proportion of sleep in mice within 30 min, which was consistent with previous studies [8, 10]. Moreover, the water extraction liquid of the herbal pair showed a stronger hypnotic effect, which indicated that the study on the combination of the two herbs is significant.

At present, there are many studies on the chemical components related to the sedative-hypnotic effect of the two herbs. The mechanisms of such effect of Semen Ziziphi spinosae have already been studied by the use of method of metabolomics [29]. However, few studies were conducted to study the sedative-hypnotic effects of their combination and the possible mechanisms of these effects.

In this study, the herbal pair Semen Ziziphi spinosae and Radix Polygalae were the research object, and the active parts of this herb pair (total flavonoids and total saponins) were acquired by D101 macroporous adsorption resin to study its sedative-hypnotic effect. The primary objective of this research was to study the sedative-hypnotic effect of this herbal pair and explore possible mechanisms. The secondary objectives were to study the sedative-hypnotic effect of the herbal pair by animal experiments, identify the main effective chemical compounds of the herbal pair, investigate the mechanisms of its sedative-hypnotic effect by measuring the levels of neurotransmitters in mice brain tissue and by methods of serum metabolomics.

## Methods

### Test drugs and instruments

Fried Semen Ziziphi spinosae (Lot: 160901) was purchased from Bozhou Jingyi Traditional Chinese Medicine Prepared Drug Pieces Factory. Radix Polygalae (Lot: 1601001) was purchased from Hebei Quantai Pharmaceutical Co., Ltd. Sodium pentobarbital (Lot: 140424) was purchased from Shanghai Ika Bio-Technology Co., Ltd. Para-chlorophenylalanine (PCPA) (Lot: C0253) was purchased from Tokyo Chemical Co., Ltd., Japan. Assay kits (201709) of 5-HT, NE, GABA, DA were purchased from Shanghai Chuangsai Technology Co., Ltd. BCA Protein Assay Kit (20170913) was purchased from Beijing Suolaibao Technology Co., Ltd. Senegenin standard (Lot: 160107) was purchased from Shanghai Ronghe Medical Technology Co., Ltd. Jujuboside A (Lot: wkq16053003; CAS: 72063–39-9) and spinosin standard (Lot: wkq16062804) were purchased from Sichuan Victory Biotechnology Co., Ltd. D101 macroporous adsorption resin was purchased from Shanghai Jinsui Biotechnology Co., Ltd.

Instruments used in this study include Versamax Microplate Reader (Miguel Molecular Instruments Shanghai Co., Ltd.), high performance liquid chromatography (Agilent, USA), ELSD6000 evaporative light scattering detector, 7890B gas chromatography (Agilent), PEGASUS HT mass spectrometer (LECO), DB-5MS column (Agilent, 30 m × 250 μm × 0.25 μm), Heraeus Fresco 17 centrifuge (Thermo Fisher Scientific), Forma 900 series ultra-low temperature refrigerator (Thermo Fisher Scientific), TNG-T98 vacuum dryer (Taicang Huamei Biochemical Instrument Factory), XHF-DY high speed disperser (Ningbo Xinzhi Biotechnology Co., Ltd.), CP24C analytical balance (Shanghai Ohaus Co., Ltd.), SC-02 low speed centrifuge (Anhui Zhongke Zhongjia Scientific Instrument Co., Ltd.), TU-1810 UV-Visible spectrophotometer (Beijing Pu Analysis General Instrument Co., Ltd.), Q Exactive Focus Orbitrap LC-MS/M (Thermo Fisher Scientific).

### Experimental animals

The Medical Ethics Committee of Shanxi University of Chinese Medicine gave ethical approval for using experimental animals for this study (Approval number: 2019LL129). All experimental procedures in this study were under the ethical standards of the Medical Ethics Committee of Shanxi University of Chinese Medicine.

Experimental animals used in this study were SPF Kunming mouse weighing 18–22 g, half male and hale female (certificate number: 0009175; license number: SCXK military 2012–0004, purchased from Experimental Animal Center of the Chinese Academy of Military Medical Sciences). Experimental mice can be used in basic experiments such as pharmacological experiments and disease model construction. All mice used in the study were euthanized by cervical dislocation.

Housing and husbandry conditions: The experimental mice were housed in small plastic cages with observable metal lids. Each cage housed six to eight mice. The cages were equipped with plastic water bottles with drinking water pipes, with purchased clean and dry mat materials were used. The mouse feed was purchased nutrient pellet feed. The housing temperature and relative humidity were kept at 18 to 22 °C and 50 to 60%, respectively. The feed and water were refilled every afternoon, and the appearances of the animals were checked each morning to determine their health status by criteria such as healthy appetite, quick response, smooth body hair, and black grain-shaped feces.

### Preparation of effective components

Fried Semen Ziziphi spinosae and Radix Polygalae were crushed into coarse powder and mixed by the mass ratio of 2:1. The mixture was degreased with petroleum ether to get the powder. The powder was then extracted twice with ten times 65% ethanol at 70 °C (each time for one hour). The extracted solution was collected and injected onto the D101 macroporous resin to separate and purify the extract.

Gradient elution was performed with distilled water 8 BV, 30 and 50% ethanol 8 BV, 70% ethanol 4 BV at a flow rate of 1 BV/h. The solid obtained by 30% ethanol eluent was used as the effective components (part) of the experiment (named EI30), and its total flavonoids and saponins were analyzed.

### Study on the sedative-hypnotic effect of EI30

Sixty mice were randomly divided into five groups (half male and half female in each group) by the aid of a random table, namely control group, Clonazepam group, EI30 high-dose group, EI30 medium-dose group, and EI30 low-dose group. The doses in the three EI30 groups were calculated by the amount of flavonoid content in the freeze-dried powder of EI30, which was 13.8 mg·kg^− 1^ for the high-dose group, 6.9 mg·kg^− 1^ for the medium-dose group and 3.45 mg·kg^− 1^ for the low-dose group. The Clonazepam group was the positive control group and was given Clonazepam at a dose of 0.01 mg·kg^− 1^. The control group was given the same amount of distilled water. Each group received gavage once per day for six consecutive days.

In the subthreshold dose sleep test, 30 min after the administration of the last dose, all animals were injected intraperitoneally with pentobarbital sodium for 25 mg·kg^− 1^. The percentage of mice falling asleep within the next 30 min was recorded.

In the suprathreshold dose sleep latency and duration test, normal gavage was performed on the seventh and eighth days, and all mice fasted for 12 h before the test. After 30 min of the last administration of treatments, 50 mg•kg^− 1^ of pentobarbital sodium was injected intraperitoneally. The sleep latency and sleep time of each mouse were recorded with the disappearance of the righting reflex as the indicator for falling asleep.

### Study on the mechanisms using PCPA induced insomnia model

#### Preparation of PCPA induced insomnia mice model

After a week of adaptive feeding, mice were intraperitoneally injected with 350 mg•kg^− 1^ PCPA once a day for three consecutive days. The animals showed a loss of circadian rhythm and were active during the day, suggesting that the insomnia model was successfully made. 32 model mice were randomly divided into 4 groups (8 mice in each group) by the aid of a random table: (1) the model control group; (2) high dose EI30 group (13.8 mg•kg^− 1^, 3) middle dose EI30 group (6.9 mg•kg^− 1^, 4) low dose EI30 group (3.45 mg•kg^− 1^). Eight normal mice were taken as the normal control group (given an equal amount of distilled water). The mice were given oral gavage once a day for 6 days.

#### Study on the mechanisms of the sedative-hypnotic effect of EI30

Before the last administration of treatments, the mice fasted for 12 h. 6 h after the last administration of treatments, brain tissues of the mice were extracted and weighed. Nine times normal saline was added to the brain tissue, which was then prepared into homogenate by high-speed disperser. The homogenate was centrifuged at 3500 r•min^− 1^ for 10 min, and the supernatant was taken to obtain 10% brain tissue homogenate. The contents of 5-hydroxytryptamine (5-HT), norepinephrine (NE), dopamine (DA), and Gamma-aminobutyric acid (GABA) in the brain tissue homogenate were determined by ELISA. The possible mechanisms of the sedative-hypnotic effect of EI30 were investigated by comparing 5-HT, DA, NE, and GABA content of the brain tissues of mice in each group.

### Analysis of effective components

#### Preparation of reference solution

Spinosin (2.3 mg) and senegenin (2.7 mg) were accurately weighed and placed in two 10-mL volumetric flasks, and volume was adjusted to with methanol and mixed well. 0.23 mg•mL^− 1^and 0.27 mg•mL^− 1^ reference solutions were obtained and were filtered with 0.45 μm microporous membrane filters before use.

#### Preparation of sample solution

43.2 mg EI30 freeze-dried powder was accurately weighed and placed in a 10-ml volumetric flask. The volume was made up to with methanol. The solution was filtered with a 0.45 μm membrane filter before used to determine the content of spinosin.

EI30 freeze-dried powder (0.271 g) was dissolved with 20 mL10% hydrochloric acid, and the solution was refluxed in a boiling water bath for 2 h to obtain brown precipitate on filter paper. Then, the filter paper was washed with a small amount of methanol several times to make the precipitate dissolved and filtered into a 10 mL volumetric flask. Methanol was used to adjust to volume. The solution was filtered with a 0.45 μm membrane filter before being used to measure the content of senegenin.

### Identification of the chemical compounds of the herbal pair

The main chemical components of the effective part EI30 were identified by LC-MS using Q Exactive Focus MS 2.5.4 by Thermo Fisher Scientific. The chromatographic separation was performed on Venusil XBP C18 column (4.6 mm × 250 mm, 5 μm) at 30 °C with a volume flow rate of 1 mL•min^− 1^ and the injection volume of 10 μL.

For spinosin [30]: The mobile phase was a gradient elution system of A (acetonitrile) and B (aqueous), and the elution was programmed as follows: 12–19% A for 0–10 min; 19–20% A for 10–16 min,; 20–100% A for 16–22 min; A 100% for 22–30 min; 100–12% A for 30–35 min. The detected wavelength was 335 nm.

For senegenin [31]: The mobile phase was a gradient elution system of A (acetonitrile) and B (0.1% phosphoric acid solution). The elution was programmed as follows: 10% A for 0–10 min; 10–65% A for 10–30 min; 65% A for 30–50 min. The detected wavelength was 210 nm.

For jujuboside A [30]: The mobile phase was a gradient elution system of A (acetonitrile) and B (aqueous). The elution was programmed as follows: 20–40% A for 0–15 min; 40% A for 15–28 min; 40–70% A for 28–30 min; 70% A-100% A for 30-32 min.

1, 3, 4, 5, and 6 μL spinosin reference solutions and 1, 2, 4, 6, 8, 10, and 20 μL senegenin reference solutions were precisely taken and analyzed under chromatographic conditions described above. Peak areas were recorded, and the standard curves using injection volume as the abscissa (X) and the peak area as the ordinate (Y) were obtained. The regression equation for spinosin was Y_1_ = 2360X_1_ + 4.8946, r_1_ = 0.9997. There was a clear linear relationship within the injection volume range of 0.2245 g to1.3542 g. The regression equation for senegenin was Y_2_ = 1279.1X_2_ + 45.453, r_2_ = 0.9999. The linear relationship was clear within the range of 0.2619 g to 5.238 g. According to the analysis, the spinosin content in EI30 was 1.61%, and the senegenin content in EI30 was 0.24%. Jujuboside A was not detected in EI30.

### Study on the mechanisms of the sedative-hypnotic effect of EI30 by methods of serum metabolomics

#### Serum metabolite extraction and derivation

Serum samples of normal group, PCPA insomnia model group, and EI30 high dose group were studied. 100 μL sample was taken from each group and put into three 1.5 mL EP tubes. Then, 0.35 mL methanol-chloroform and 20 μL of L-2-Chlorophenylalanine (1 mg/mL stock in dH_2_O) were added to the EP tubes. The samples were swirled for 30s mix thoroughly, after which they were kept at − 20 °C for 10 min before being centrifuged for 15 min with a speed of 12,000 rpm, at 4 °C. Subsequently, 0.4 mL supernate from each EP tube was transferred into 2 mL injection vials. 15 μL from each sample was taken and pooled as the quality control (QC) sample.

All the above samples in injection vials were dried entirely in a vacuum concentrator without heating. Then, 60 μL methoxymethyl amine salt (dissolved in pyridine, with a final concentration of 20 mg/mL) was added, and the mixtures were incubated for 30 min at 80 °C. After that, 80 μL of the BSTFA regent (containing 1% tetrachloro-4-methylsulfonyl, vol/vol) was added into the mixture, which was then being incubated for 1.5 h at 70 °C. Later, 10 μL FAMEs (Standard mixture of fatty acid methyl esters, 1 mg/mL stock in chloroform C8-C16) was added. After cooling to room temperature, all samples were analyzed by GC-TOF-MS [32].

#### GC-TOF-MS data collection on serum metabolite

GC-TOF-MS analysis was performed using an Agilent 7890 gas chromatograph system coupled with a Pegasus HT time-of-flight mass spectrometer (GC-TOF-MS), which used a DB-5MS capillary column (30 m × 250 μm inner diameter, 0.25 μm film thickness, J&W Scientific, Folsom, CA, USA) [32]. The specific analysis conditions of GC-TOF-MS were as follows: Injection volume: 1 μL; Mode: splitless; Front inlet purge flow: 3 mL•min^− 1^; Gas flow rate through column: 1 mL•min^− 1^; Temperature: initially kept at 50 °C for 1 min, then raised to 310 °C at a rate of 20 °C/min and kept at 310 °C for 6 min; Injection temperature: 280 °C; Transfer line temperature: 280 °C; Ion source temperature: 250 °C; Ionization voltage: − 70 ev; Scanning mode: 50–500 m/z; Scanning rate: 20 spectra per second; Solvent delay: 366 s.

#### Mass spectrometer (MS) data processing and analysis

The MS data were put into Chroma TOF 4.3X software (LECO Corporation) for analysis, such as peak detection, baselines correlation, deconvolution analysis, and peak list alignment. For metabolites identification, the LECO-Fiehn database (library for mass spectrum and retention index) was used. Peaks with the detection rate of less than 50%, or relative standard deviation (RSD) > 30% in the QC sample were removed. The raw data were preprocessed, and only the peak area data with missing values less than 50% in all groups were retained. Then the missing values were filled with 1/2 of the minimum value. Finally, the internal standard method was used to normalize the data for statistical analysis.

The processed data were imported into SIMCA14.1 software for Principal Component Analysis (PCA), which revealed the distribution of metabolite in mice serum samples. To further investigate separations between groups and which variables were account for the separations (identify effective biomarkers), Orthogonal Projections to Latent Structures discriminant analysis (OPLS-DA) was performed to get score scatter plots between groups. Permutation tests were used to verify the validity of the OPLS-DA model. R^2^ and Q^2^ were used to express the validity of the OPLS-DA model. Variable importance projection (VIP) value of the first principle component, together with results of Student’s *t*-test were obtained to determine differential metabolites that are significant. Variables with high VIP values (> 1.0) and statistical significance (*P* < 0.05) were identified as potential differential metabolites [33, 34]. Those potential differential metabolites were also visualized by volcanic maps. The metabolic pathways related to those identified differential metabolites were predicted and explored by MetaboAnalyst 3.0 (Http://www.metaboanalyst.ca/) and databases KEGG (https://www.genome.jp/kegg/).

## Results

### Sedative-hypnotic effect of EI30

All the animals in the control group, Clonazepam group and each EI30 group were included in the data analysis. SPSS13.0 was used for analysis, and according to the type the data, Fisher’s exact tests were performed to compare the proportion of mice sleeping within 30 min between the control groups and each EI30 group. As shown in Table 1, compared with the control group, the EI30 high-dose group and the Clonazepam group were with significantly higher proportions of mice sleeping within 30 min (*P* = 0.027 and *P* = 0.005 respectively). Medium-dose and low-dose groups showed relatively weaker effects (*P* > 0.05).

**Table 1:** Effect of EI30 on the proportion of mice sleeping within 30 min

Group	Dosage /mg·kg^− 1^	Number of mice	Number of sleeping within 30 min	Proportion of mice sleeping within 30 min /%
Control group	–	12	5	42
Clonazepam	0.01	12	11	92*
EI30 high-dose group	13.8	12	11	92*
EI30 medium-dose group	6.9	12	10	83
EI30 low-dose group	3.45	12	10	83

Note: * compared with the control group, *P* < 0.05

Student’s *t-*tests were performed to compare sleep latency and duration between the control groups and each EI30 group. Results in Table 2 shows that compared with the control group, the Clonazepam group, and all three EI30 groups were with significantly shorter sleep latency (*P* < 0.01) and longer sleeping duration (*P* < 0.01).

**Table 2:** Effect of EI30 on sleep latency and duration ($\overline{x}$ ±s)

Group	Dosage/(mg·kg^− 1^)	Sleep latency /min	Sleep duration/min
Control group	–	41.2 ± 19.8	2.8 ± 3.1
Clonazepam	0.01	2.78 ± 0.83**	142.5 ± 13.44**
EI30 high-dose group	13.8	4.5 ± 2.4**^##^	18.7 ± 11.7**
EI30 medium-dose group	6.9	4.0 ± 1.9**^##^	10.1 ± 4.5**
EI30 low-dose group	3.45	6.8 ± 1.9**^##^	8.6 ± 2.4**

Note:** compared with the control group, *P* < 0.01. ^##^ compared with the active control group, *P* < 0.01

### Effect of EI30 on 5-HT, NE, DA, and GABA in brain tissue of mice

PCPA is a synthetic inhibitor of 5-HT. Compared with the normal group, the content of 5-HT in total protein in brain tissue of mice in the model group was significantly lower, and the content of excitatory neurotransmitters NE and DA in total protein was higher in the model group, suggesting the models were produced successfully (As can be seen in Table 3 and Table 4).

**Table 3:** Contents of neurotransmitters 5-HT and GABA in brain tissues of mice (*n* = 10, $\overline{x}$ ±s)

Group	Dosage/mg•kg^−1^	5-HT/10^−2^	GABA/nmol•ng^− 1^
Normal group	–	3.91 ± 0.73	1.22 ± 0.24
Model group	–	2.58 ± 0.20^**^	1.77 ± 0.18^**^
EI30 high-dose group	13.8	3.16 ± 0.39^△△^	1.52 ± 0.18^*△^
EI30 medium-dose group	6.9	3.37 ± 0.56^△△^	2.00 ± 0.32^**^
EI30 low-dose group	3.45	3.70 ± 0.62^△△^	2.03 ± 0.19^**△^

Note: Compared with the normal group: * *P* < 0.05, ** *P* < 0.01. Compared with the model group: ^△^*P*<0.05, ^△△^*P*<0.01

**Table 4:** Contents of excitatory neurotransmitters NE and DA in brain tissues of mice (*n* = 10, $\overline{x}$ ±s)

Group	Dosage/mg•kg^−1^	NE/10^−3^	DA/10^−3^
Normal group	–	10.95 ± 1.01	6.34 ± 0.67
Model group	–	18.90 ± 1.86^**^	11.25 ± 2.26^**^
EI30 high-dose group	13.8	15.52 ± 1.27^**^	10.24 ± 1.23^**^
EI30 medium-dose group	6.9	17.42 ± 0.94^**^	8.20 ± 0.77^**△^
EI30 low-dose group	3.45	17.75 ± 2.14^**△△^	9.18 ± 1.09^**^

Note: Compared with the normal group: * *P* < 0.05, ** *P* < 0.01.Compared with the model group: ^△^*P*<0.05, ^△△^*P*<0.01

Tables 3 and 4 demonstrate the contents of neurotransmitters 5-HT, GABA, NE, and DA in the brain tissues of mice. Compared with the model group, the content of 5-HT in the total protein in brain tissue of mice was significantly higher in all the three EI30 groups (*P* < 0.01). The content of NE in the total protein was significantly lower in the low-dose EI30 group (*P* < 0.01), and the content of DA in the total protein was significantly lower in the medium-dose group (*P* < 0.05). The content of GABA was significantly lower for the high-dose group (*P* < 0.05).

### Identified compounds by LC-MS

Eleven main compounds were identified in EI30 (the effective components of the herbal pair) by LC-MS. The results are shown in Table 5.

**Table 5:** Information of Identified compounds in EI30 by LC-MS

No.	t_R/min_	Molecular Formula	Selected ion	Experimental	Theoretical	Identification
1	4.74	C_23_H_29_ NO_8_	[M + H]^+^	448.1981	448.1966	6-glc-coclaurine^b^
2	5.93	C_19_H_24_NO_3_^+^	M^+^	314.1746	314.1751	Magnocurarine^a^
3	5.27	C_16_H_19_NO_8_	[M-H]^−^	353.1043	352.1038	N-glc-indoleaceticacid^b^
4	7.41	C_20_H_24_NO_4_^+^	M^+^	342.1697	342.1700	Zizyphusine
5	7.65	C_23_H_32_O_15_	[M-H]^−^	547.1674	547.1668	Sibiricoses A6
6	9.31	C_24_H_26_O_14_	[M-H]^−^	537.1257	537.1250	Sibiricaxanthone A
7	10.89	C_25_H_28_O_15_	[M-H]^−^	567.1363	567.1355	Polygalaxanthone III
8	12.10	C_28_H_32_O_15_	[M-H]^−^	607.1679	607.1668	Spinosin
9	21.70	C_34_H_42_O_19_	[M-H]^−^	753.2261	753.2247	3,6′-Disinapoylsucrose.
10	22.91	C_38_H_40_O_18_	[M + H]^+^	785.2288	785.2287	6″’-Feruloylspinosin
11	25.44	C_31_H_38_O_17_	[M-H]^−^	681.2050	681.2036	Tenuifoliside A

### ***GC-TOF-MS results*** of all serum samples and results of PCA

**Fig. 1:**
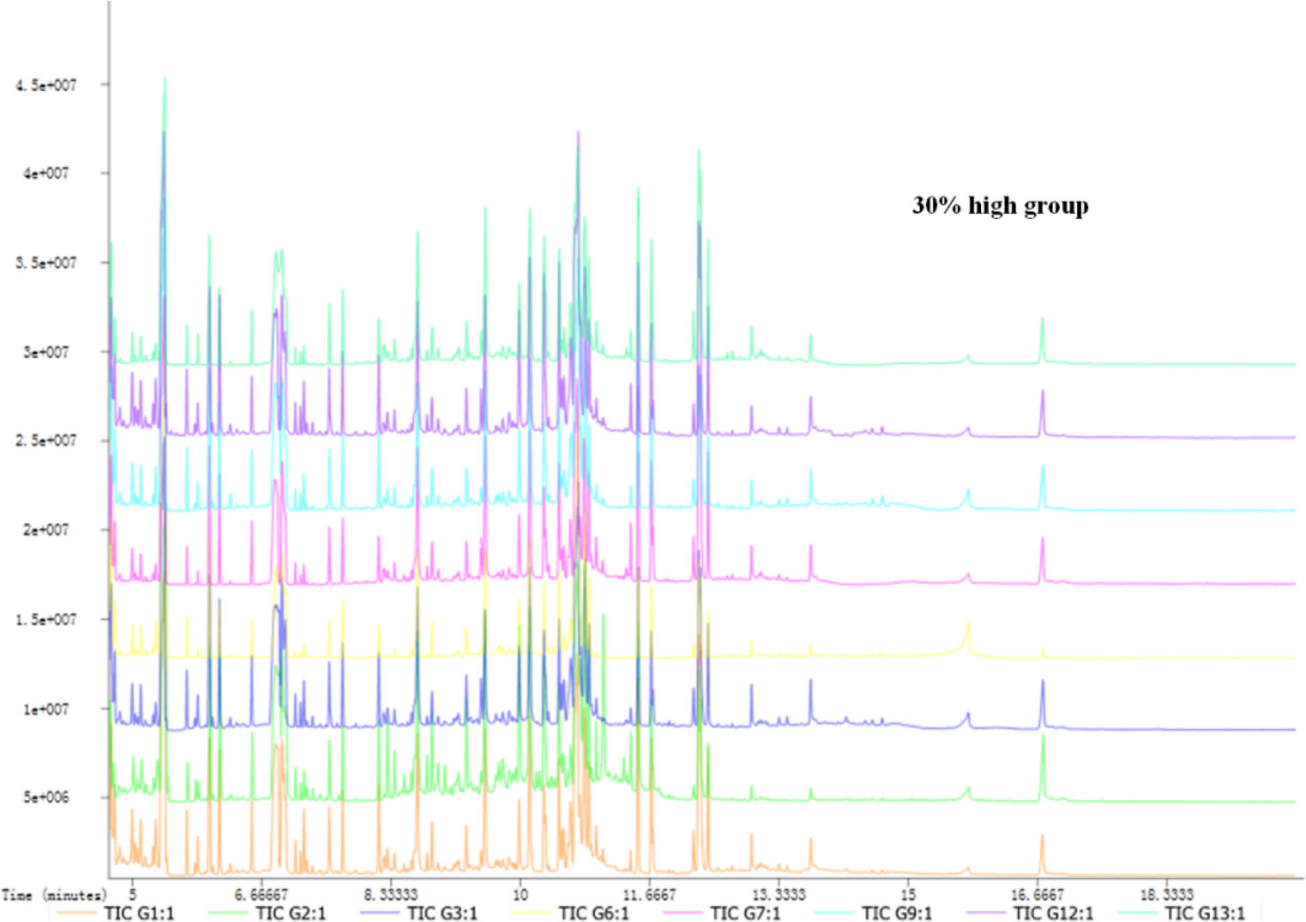
GC-MS of serum samples for normal group, model group, and EI30 high-dose group (30% high group)

GC-TOF-MS results of serum samples for normal group, model group, and EI30 high-dose group are shown in Fig. 1.

**Fig. 2:**
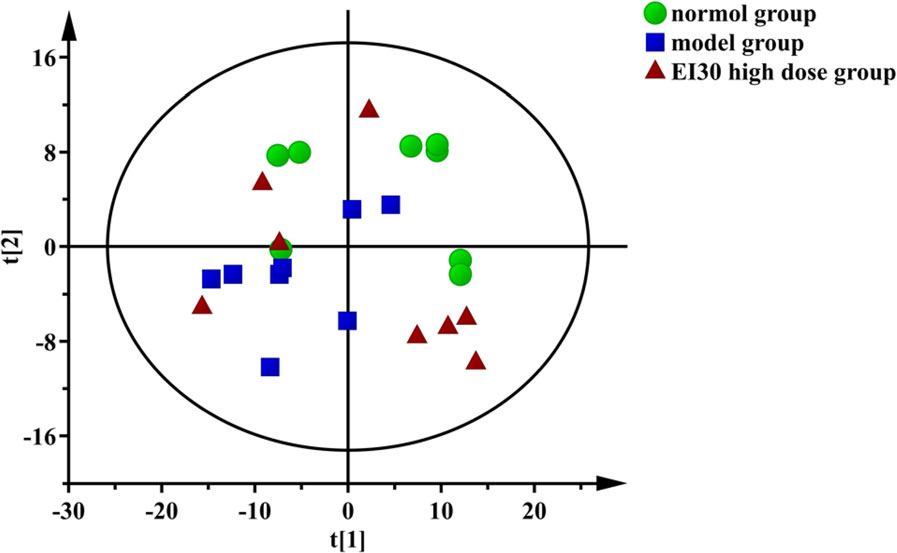
Scatter plot of PCA for all three samples

Based on GC-MS results of different groups’ serum samples, PCA, an unsupervised multivariable data analysis method, were first used to comprehensively analyze and visualize data from all experiment groups [35]. Results of PCA are shown in Fig. 2. Abscissa t[1] and ordinate t[2] represent the scores of the principal components ranking first and second, respectively. Scatters with different colors and shapes represent different sample groups. As can be seen from Fig. 2, the results of all samples were basically within the Hoteling’s T^2^ ellipse (95%). However, there were no distinct differences between any of the two groups, and this is because of the characteristics of the GC-TOF-MS metabolite profiling data were of a large number of metabolites but coming from relatively small samples [36]. Among these variables, there were not only differential variables but also undifferentiated variables. As a result, in the PCA model, the differential variables were not separated and visualized well, and therefore cannot be further interpreted. The results of PCA need to be further studied.

### Results of OPLS-DA

**Fig. 3:**
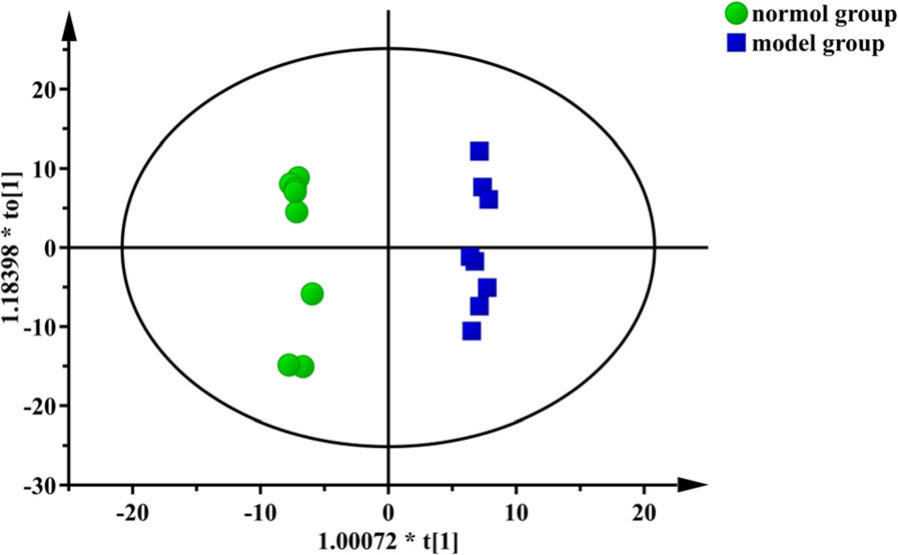
OPLS-DA score scatter plot for the normal group and the model group

**Fig. 4:**
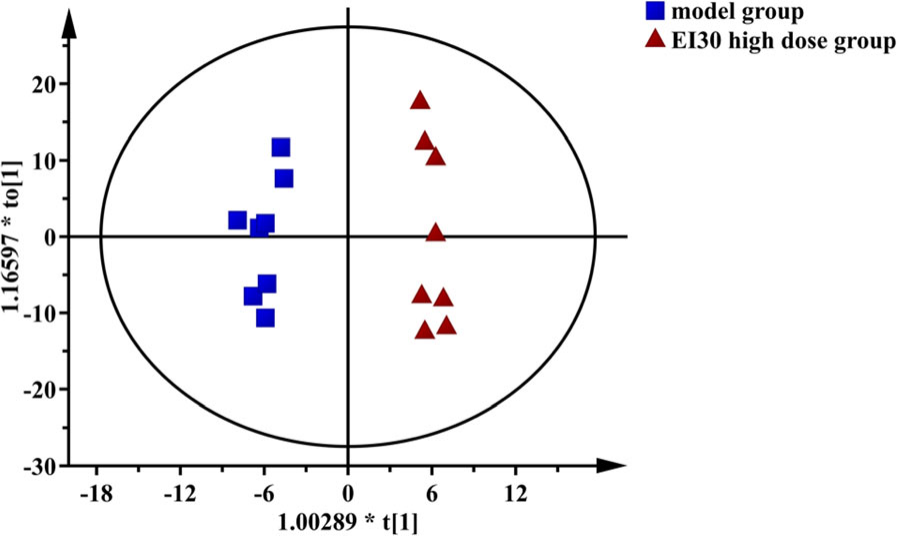
OPLS-DA score scatter plot for the model group and EI30 high-dose group

**Fig. 5:**
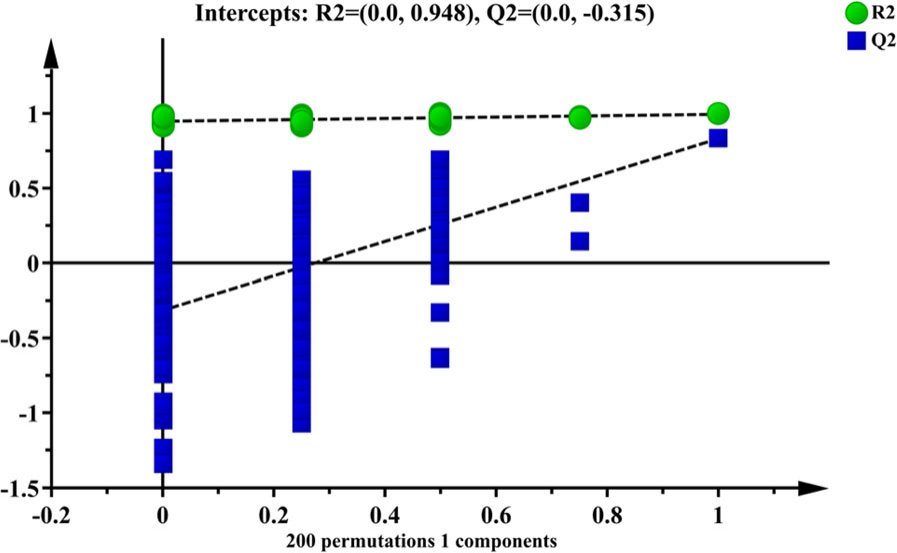
OPLS-DA permutation test result between the normal group and the model group

**Fig. 6:**
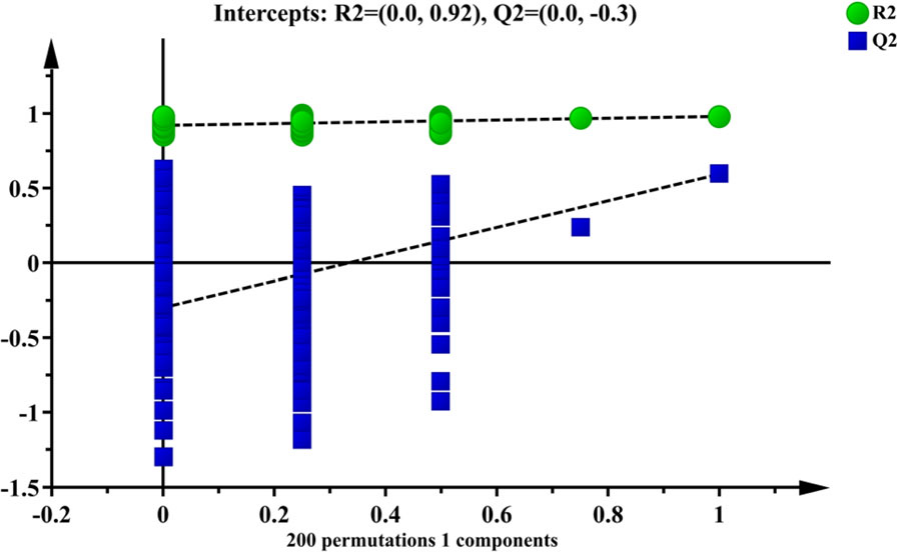
OPLS-DA permutation test result between the model group and EI30 high dose group

To find out the relevant variables reflecting differences between groups, the OPLS-DA model was subsequently used [37]. The results are shown in Figs. 3, 4, 5 and 6. Figures 3 and 4 are score scatter plots of the analysis results between the normal group and the model group and between the model group and EI30 high-dose group. In these two figures, t[1] and t_0_[1] respectively represent the score of the first predictive principal component and the score of the orthogonal principal component, which captures variations between groups. Scatters of different shapes and colors represent different sample groups. As can be seen from the two figures, there were systematic differences in metabolites between the normal group and the model group, and between the model group and EI30 high-dose group, indicating that first the PCPA induced insomnia mice model was successfully made, and EI30 had obvious effects on the model [38]. Compared with the results of PCA, the OPLS-DA model showed the differences between sample groups more clearly. Thus in this study, OPLS-DA provided a better way to demonstrate the differences in serum metabolites between groups [39].

Figures 5 and 6 are the results of the permutation tests from OPLS-DA between different groups. The values of the two parameters R2 and Q2, reflect the quality and reliability of the computed OPLS-DA model. R2 represents the goodness of fit, and Q2 represents the predictive ability of the model. The closer the two values are to 1, the better the OPLS-DA model is in explaining the differences between groups [40].

The horizontal coordinate in the two figures represents the correlation coefficient to original values, and the vertical coordinate represents R2 and Q2 values. Green dots and blue squares represent the R2 and Q2 scores obtained by the permutation test. The two dotted lines represent regression lines for R2 and Q2. The intercept R2 in Figs. 5 and 6 were 0.984 and 0.92, respectively, and the intercept Q2 in Figs. 5 and 6 were − 0.315 and − 0.3, respectively. The results showed that the OPLS-DA model was robust in reflecting the differences between groups, and overfitting was not a problem in this model.

### Results of differential metabolites and analysis

**Fig. 7:**
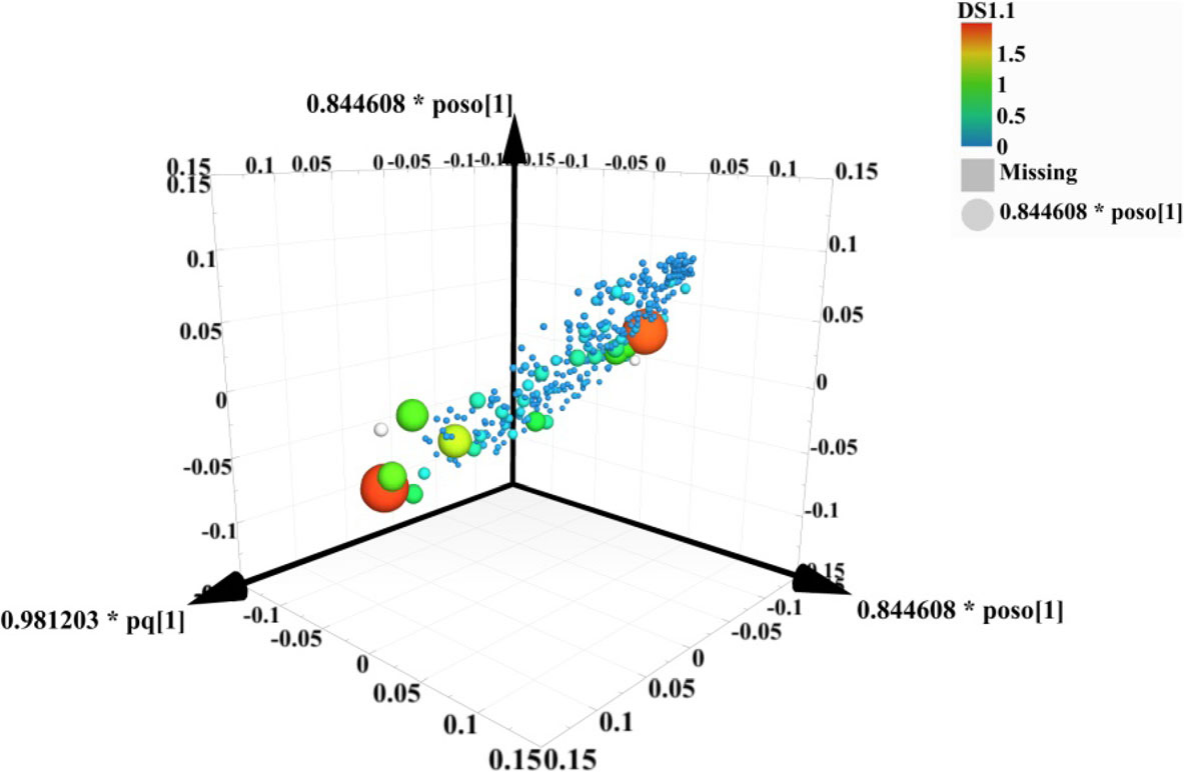
Volcano plot of differential metabolites between the normal group and the model group

**Fig. 8:**
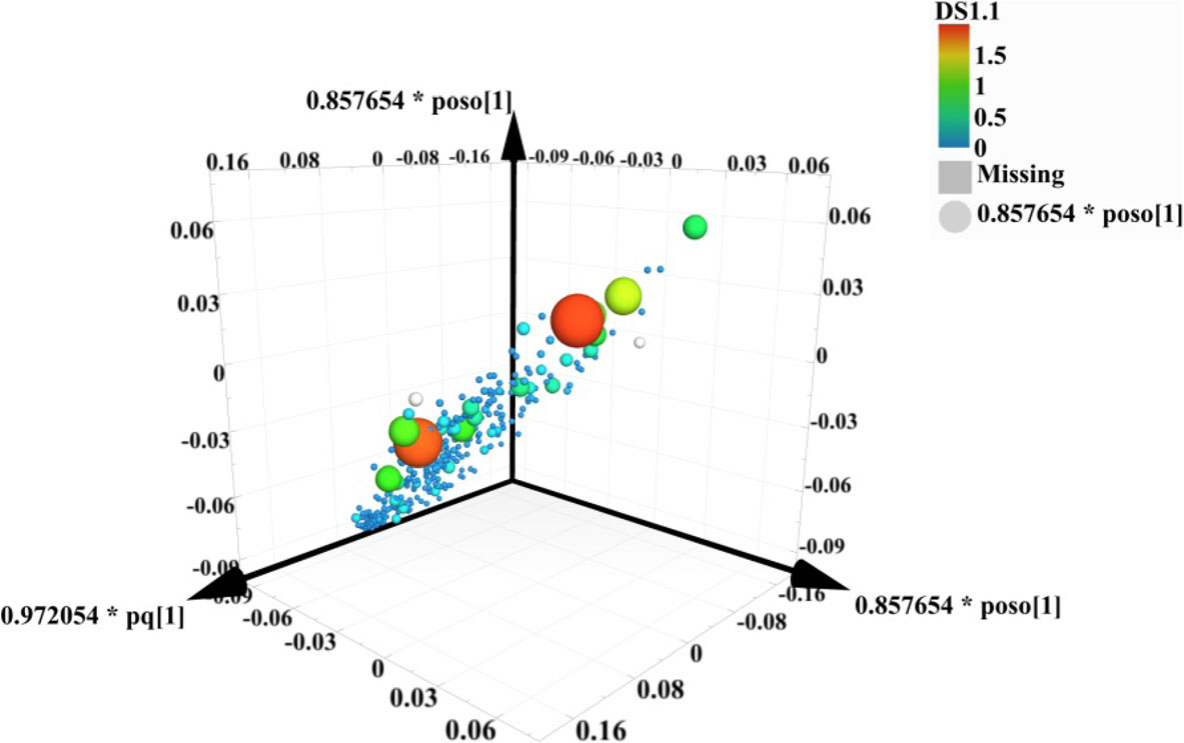
Volcano plot of differential metabolites between the model group and EI30 high-dose group

As shown in Figs. 7 and 8, differential metabolites found by the analysis were summarized by volcano plots. Each point in the scatter plots represents a metabolite: The size of the points represents the VIP value in the OPLS-DA model; The color of the points indicates whether the metabolites were significantly different between the two groups. Red indicates that the level of the metabolite was significantly higher in the model group compared with the normal group, and in the EI30 high-dose group compared with the model group; blue indicates the level of the metabolite was significantly lower; gray represents metabolites without statistically significant differential levels [35, 41].

The differential metabolites between the normal group and the model group, and between the EI30 high-dose group and the model group were selected from OPLS-DA with VIP>1 and *P* < 0.05 (See supplementary materials Table S1-S2). Table 6 illustrates that in total, 17 common differential metabolites with significance were found between the normal group and model group, and between the model group and EI30 high-dose group. It is interesting to notice that the downregulation and upregulation of the 17 common differential metabolites showed regularities.

**Table 6:** Common differential metabolites and variations

NO.	biomarkers	模型组^a^	高剂量组^b^
1	beta-Mannosylglycerate 2	↓	↑
2	Myristic Acid	↓	↑
3	lauric acid	↓	↑
4	palmitoleic acid	↓	↑
5	1-Monopalmitin	↓	↑
6	5,6-dihydrouracil 1	↓	↑
7	palmitic acid	↓	↑
8	pantothenic acid	↓	↑
9	panthenol 2	↓	↑
10	phthalic acid	↑	↓
11	pentadecanoic acid	↓	↑
12	Isoleucine	↓	↑
13	piceatannol 2	↓	↑
14	trans-4-hydroxy-L-proline 2	↑	↓
15	phenylacetaldehyde 2	↓	↑
16	D-Altrose 1	↓	↑
17	Sorbitol	↓	↑

(The ↑↓variations of differential metabolites for the model group column are results compared with normal group, and the ↑↓variations for EI30 high dose group are results compared with model group)

Among the 17 common differential metabolites, 15 of them, such as beta-mannosylglycerate 2, myristic acid, lauric acid, palmitoleic acid, 1-monopalmitin, were downregulated in the model group when compared with the normal group but upregulated in the EI30 high-dose group when compared with the model group. On the contrary, the other two differential metabolites, phthalic acid and trans-4-hydroxy-L-proline, were upregulated in the model group compared with the normal group but downregulated after the high-dose EI30 intervention.

### Analyses and results of metabolic pathways

The differential metabolites between the normal group and model group and the differential metabolites between the model group and EI30 high-dose group (See supplementary materials Table S1-S2) were used for analyzing related metabolic pathways. Online analysis platform MetaboAnalyst3.0 (Http://www.metaboanalyst.ca/) was used to analyze the relevant metabolic pathways. *Mus musculus* (KEGG) was used as the database. The identified pathways went through enrichment analysis (using hypergeometric distribution) and topology analysis (using relative-betweenness centrality). All metabolic pathways found displayed their impact values and *P*-values in the form of bubbles.

**Fig. 9:**
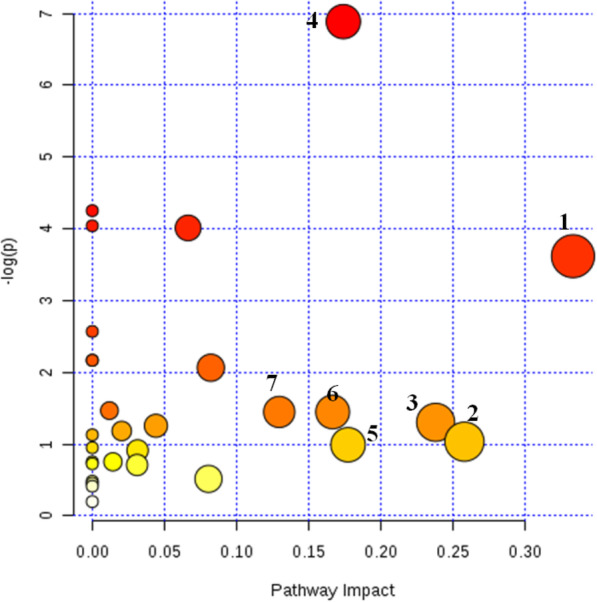
Metabolic pathways for differential metabolites between the model group and normal group. (“1” biosynthesis of valine, leucine, and isoleucine; “2” metabolism of glyoxylic acid and dicarboxylic acid; “3” metabolism of nicotine and niacinamide; “4” tricarboxylic acid cycle; “5” metabolism of starch sucrose; “6”metabolism of riboflavin; “7” metabolism of phenylalanine)

**Fig. 10:**
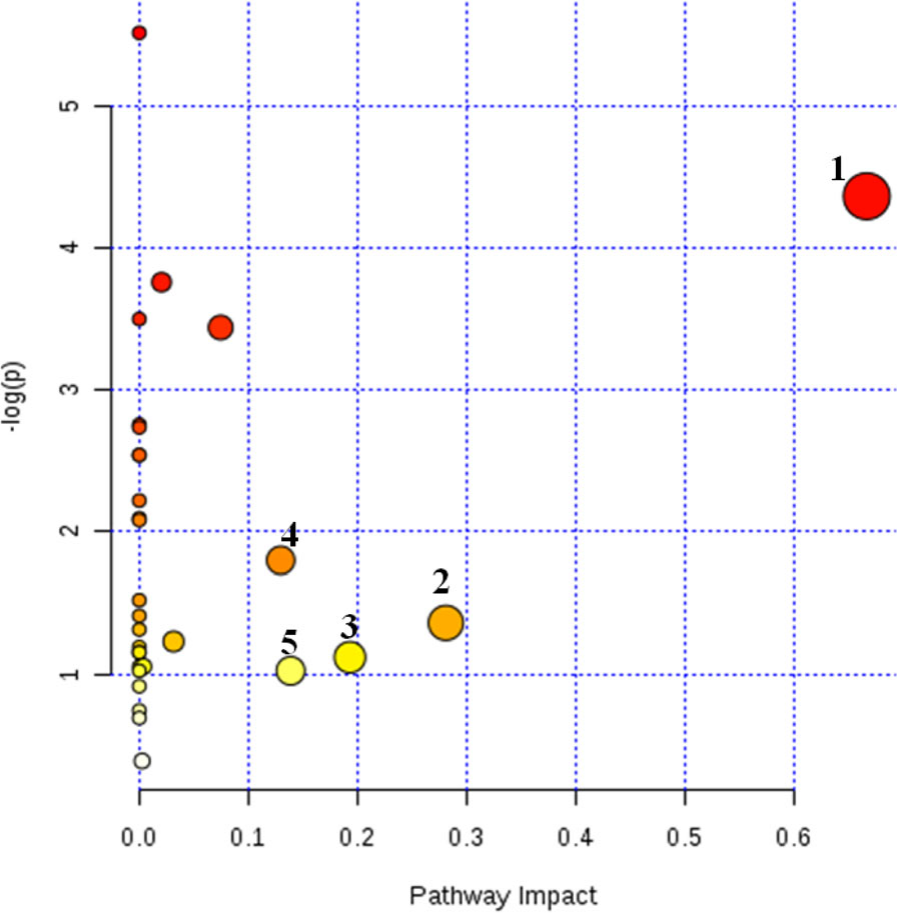
Metabolic pathways for differential metabolites between EI30 high-dose group and model group. (“1” biosynthesis of valine, leucine, and isoleucine; “2” metabolism of glyceride; “3” metabolism of alanine, aspartic acid, and glutamic acid; “4” metabolism of phenylalanine; “5” metabolism of cysteine and methionine)

Figures 9 and 10 are the results of metabolic pathways found for the differential metabolites between the model group and the normal group, and between EI30 high-dose group and the model group, respectively. Every bubble in these figures represents a metabolic pathway. The size of the bubble and its position on the horizontal axis (represent the impact of pathways) shows the impact factor in topology analysis. The vertical coordinate and the color of the bubbles represent the *P*-value in the enrichment analysis. The darker the color, the smaller the *P*-value is. The cut-off value of the impact factor was set as 0.1, and pathways of greater or equal value to this would be considered as relevant target pathways.

According to the analysis results in Fig. 9, seven relevant metabolic pathways were involved in PCPA induced insomnia in mice, which were biosynthesis of valine, leucine and isoleucine, metabolism of glyoxylic acid and dicarboxylic acid, metabolism of nicotine and niacinamide, tricarboxylic acid cycle, metabolism of starch sucrose, metabolism of riboflavin, and metabolism of phenylalanine. Based on the analysis results from Fig. 10, the sedative-hypnotic effect of EI30 on PCPA induced insomnia in mice were related to five metabolic pathways, biosynthesis of valine, leucine, and isoleucine, metabolism of glyceride, metabolism of alanine, aspartic acid and glutamic acid, metabolism of phenylalanine, and metabolism of cysteine and methionine.

## Discussion

### Sedative-hypnotic effect of EI30

Semen Ziziphi spinosae and Radix Polygalae are two herbs commonly used for sedative and hypnotic purposes in traditional Chinese medicine, and they are often used together as a pair [42]. In this research, we aimed to study the sedative-hypnotic effect of this herbal pair and investigate its possible mechanism by studying EI30, the effective components of the herbal pair. The results showed that high-dose EI30 significantly increased the proportion of sleeping within 30 min, and high, medium, and low-dose EI30 can significantly shorten sleep latency of pentobarbital sodium-induced sleep and prolong sleeping time, indicating that EI30 could promote sleep in mice, although the effect is still weaker when compared with Clonazepam. The results showed that the herbal pair had sedative-hypnotic effects, providing a research basis for the future development and utilization of this herbal pair.

### Mechanism of the sedative-hypnotic effect of EI30 on the treatment of insomnia

PCPA, a synthetic inhibitor of neurotransmitter 5-HT, can cause the disappearance of circadian rhythm and lead to insomnia, and it is well recognized to be used to create insomnia model [43]. In this experiment, mice were intraperitoneally injected with PCPA for three consecutive days, and after injection, the mice showed increased activity and sensitivity to sounds, the disappearance of circadian rhythm, and restlessness during the day, indicating successful model creation.

5-HT is one of the vital neurotransmitters in the central nervous system, and an increase of 5-HT in the brain can encourage the occurrence of sleep [44]. The results of this study showed that EI30 could increase the proportion of 5-HT in the total protein in brain tissue in mice, suggesting that the sedative and hypnotic effect of EI30 is related to an increased level of 5-HT in the brain protein.

Neurotransmitter NE is mainly responsible for the central nervous excitement and awakening function, and it is related to fast wave sleep and can regulate the sleep-wake cycle [45]. The results in this study showed that EI30 reduced the proportion of NE in total protein in mouse brain tissue, showing that the sedative-hypnotic effect of EI30 is related to the reduction of NE in brain protein.

GABA is an important inhibitory neurotransmitter in the central nervous system [46, 47]. Increased level of GABA in the brain is associated with alleviation of anxiety, and it is of sedative-hypnotic effect [48, 49]. However, findings of current studies on the changes in GABA level in the brain of insomnia models are divided. It is not clear whether sleep deprivation is associated with an increased or decreased level of GABA. The results of this study also showed inconsistent results. The content of GABA was significantly higher for the model group compared with the normal group, and EI30 high-dose group had a significantly lower level of GABA compared with the model group, while the EI30 low-dose group showed a significantly higher level of GABA compared with the model group.

Another neurotransmitter DA also plays an important role in wakefulness. The excitation of DA neurons is related to an increased level of wakefulness [50]. The results of this study showed the effect of EI30 is also associated with the reduction of DA in the brain.

The results of this study showed that EI30 has a good sedative-hypnotic effect. This effect is possibly related to the adjustment of 5-HT, NE, GABA, and DA levels in mice brain.

### Effective components analysis

Spinosin [13, 51] and jujuboside A [14] are the main monomer compounds of sedative-hypnotic effect in Semen Ziziphi spinosae. Radix Polygalae contains senegenin. Contents of spinosin, jujuboside A, and senegenin in EI30 freeze-dried powder were determined in this study. The results showed that the contents of spinosin and senegenin were 1.61 and 0.61%, respectively, but no jujuboside A was detected in EI30, as there was no spot at the corresponding positions of jujuboside A in TLC (Thin layer chromatography). However, in our study, other effective parts (solid obtained by 50% ethanol and 70% ethanol in the gradient elution) were named EI50 and EI70. For EI50 and EI70, there were spots at the corresponding positions of jujuboside A, showing that jujuboside A was absent in EI30, but enriched in other effective parts like EI50 and EI70.

### Discussion on metabolism mechanisms of EI30 sedative-hypnotic effect in vivo

GC-MS is one of the most commonly used methods for metabolite analysis of biological samples in disease diagnosis and biomarker identification [52]. In this study, GC-TOF-MS, combined with multivariate analysis, were used to study the changes in serum metabolites of PCPA induced insomnia mice model under EI30 intervention.

By selecting results from OPLS-DA (VIP > 1 and *P* < 0.05), in total, 17 common differential metabolites were identified between the normal group and model group, and between the model group and EI30 high-dose group. They were beta-Mannosylglycerate 2, palmitoleic acid, phthalic acid, 1-Monopalmitin, inosine, and pantothenic acid. Five of them, beta-Mannosylglycerate 2, palmitoleic acid, 1-Monopalmitin, inosine, and pantothenic acid, were downregulated in the model group compared with the normal group but upregulated in the EI30 high-dose group compared with the model group. The other two, phthalic acid and trans-4-hydroxy-L-proline, were upregulated in the model group compared with the normal group and downregulated in EI30 high-dose group compared with the model group. The upregulation and downregulation of the common differential metabolites showed the successful production of the PCPA induced mice model of insomnia and the effectiveness of the metabolic regulation of high-dose EI30 intervention.

The results of metabolic pathway analysis showed that the seven metabolic pathways involved in mice insomnia induced by PCPA were biosynthesis of valine, leucine, and isoleucine, metabolism of glyoxylic acid and dicarboxylic acid, metabolism of nicotine and niacinamide, tricarboxylic acid cycle, metabolism of starch sucrose, metabolism of riboflavin, and metabolism of phenylalanine. The sedative-hypnotic effects of EI30 on PCPA induced insomnia in mice were related to five metabolic pathways, biosynthesis of valine, leucine, and isoleucine, metabolism of glyceride, metabolism of alanine, aspartic acid, and glutamic acid, metabolism of phenylalanine, and metabolism of cysteine and methionine.

This study showed that two metabolic pathways were involved in metabolic regulations of both insomnia and the EI30 intervention, which were biosynthesis of valine, leucine, and isoleucine and metabolism of phenylalanine. Findings from a previous study showed that in the PCPA insomnia model, the biosynthetic pathway of valine, leucine and, isoleucine was upregulated, and after proper drug intervention, this metabolic pathway was tending to be normal [53]. The similar results were obtained in this study.

Valine, leucine, and isoleucine are essential amino acids for the human body, which are involved in the stress response, energy metabolism, and muscle metabolism process [54]. Valine is of neuroprotective effect, and leucine is essential in muscle metabolism [54]. Leucine deficiency can lead to muscle tremors [54]. Therefore, the PCPA induced insomnia model is possibly related to nerve injury and increased glucose metabolism. The results of this study showed that EI30 has a regulatory effect on the abnormal metabolism of leucine and isoleucine caused by PCPA and can make the metabolism of valine, leucine, and isoleucine tend to be normal.

Phenylalanine can produce tyrosine via phenylalanine hydroxylase, and tyrosine is essential for the production of neurotransmitter DA [55, 56]. Dopamine is essential for the production of NE [57]. DA and NE are the key neurotransmitters that cause insomnia. The results of serum metabolomics in this study showed that PCPA could lead to abnormal phenylalanine metabolism. The EI30 intervention downregulated the production of phenylalanine. There were previous serum metabolomics findings that the sedative-hypnotic effect of Semen Ziziphi spinosae was related to correcting phenylalanine metabolism disorder caused by PCPA [39]. The findings in this study showed that the levels of DA and NE in the model group were significantly higher compared with the normal group, but in the EI30 intervention group, the levels of both were decreased. Based on these findings, it is speculated that PCPA is causing insomnia by inhibiting the synthesis of 5-HT, disrupting the metabolism of monoamine neurotransmitters. While EI30 may play a sedative-hypnotic role by regulating the metabolism of phenylalanine, thereby inhibiting the synthesis of excitatory neurotransmitter DA and NE.

## Conclusions

This study evaluated the sedative-hypnotic effect of herbal pair Semen Ziziphi spinosae and Radix Polygalae by animal experiments, and the main chemical compounds in the effective components of herbal pair were identified. The results of this study identified the possible mechanisms of insomnia induced by PCPA and biomarkers helpful for the early diagnosis. Also, the study was the first to preliminary investigate the metabolism mechanisms of the sedative-hypnotic effect of the combination of Semen Ziziphi spinosae and Radix Polygalae by metabolomics approach, providing a research basis for the future development and utilization of this herbal pair in the treatment of insomnia.

## Supplementary information

**Additional file 1: Table S1-S2 **

## Data Availability

The datasets used and/or analysed during the current study are available from the corresponding author on reasonable request.

## Abbreviations

5-HT: 5-hydroxytryptamine
DA: dopamine
GABA: gamma-aminobutyric acid
GC-TOF-MS: gas chromatography time-of-flight mass spectrometry
LC-MS: liquid chromatography–mass spectrometry
NE: norepinephrine
OPLS-DA: orthogonal projections to latent structures discriminant analysis
PCA: principal components analysis
PCPA: para-chlorophenylalanine
QC: quality control
