# Supplementary material for: Revealing the sedative-hypnotic effect of the extracts of herb pair Semen Ziziphi spinosae and Radix Polygalae and related mechanisms through experiments and metabolomics approach
Source: BMC Complement Med Ther. 2020 Jul 2;20:206. doi: 10.1186/s12906-020-03000-8 (PMC7330955; doi:10.1186/s12906-020-03000-8)
Supplement: Supplementary file 1 — Additional file 1: Table S1-S2 [file 12906_2020_3000_MOESM1_ESM.docx]

**Supplementary materials**

**Table S1. Differential** **metabolites between normal group and model group**

| chemical compound | Sim. | R.T.  /min | m/z | Normalized Area | | VIP | P | Tre. |
| --- | --- | --- | --- | --- | --- | --- | --- | --- |
|  |  |  |  | CON | MOD |  |  |  |
| succinic acid | 941 | 7.25 | 147 | 0.224 | 0.071 | 2.079 | 2.92E-05 | ↓ |
| sorbitol | 850 | 10.91 | 333 | 0.015 | 0.006 | 2.003 | 0.001 | ↓ |
| beta-Mannosylglycerate 2 | 968 | 10.50 | 217 | 0.206 | 0.010 | 1.979 | 0.001 | ↓ |
| tyrosine 1 | 886 | 10.98 | 218 | 0.365 | 0.176 | 1.964 | 0.001 | ↓ |
| palmitoleic acid | 913 | 11.42 | 117 | 0.068 | 0.015 | 1.917 | 0.002 | ↓ |
| pantothenic acid | 714 | 11.22 | 291 | 0.002 | 0.001 | 1.857 | 0.001 | ↓ |
| Glutaric Acid | 544 | 7.87 | 261 | 0.007 | 0.002 | 1.849 | 0.001 | ↓ |
| guanine 2 | 242 | 11.36 | 366 | 0.001 | 0.0003 | 1.837 | 0.002 | ↓ |
| tryptophan 2 | 164 | 12.26 | 314 | 0.003 | 0.001 | 1.834 | 0.003 | ↓ |
| palmitic acid | 947 | 11.52 | 117 | 1.694 | 0.964 | 1.812 | 0.002 | ↓ |
| pentadecanoic acid | 699 | 11.03 | 132 | 0.004 | 0.001 | 1.809 | 0.002 | ↓ |
| panthenol 2 | 241 | 11.04 | 71 | 0.03 | 0.013 | 1.785 | 0.002 | ↓ |
| 5,6-dihydrouracil 1 | 567 | 8.46 | 243 | 0.002 | 0.001 | 1.784 | 0.001 | ↓ |
| Isoleucine | 944 | 7.10 | 158 | 0.22 | 0.113 | 1.715 | 0.002 | ↓ |
| 1-Monopalmitin | 805 | 13.79 | 371 | 0.003 | 0.002 | 1.665 | 0.01 | ↓ |
| D-Altrose 1 | 652 | 10.77 | 273 | 0.043 | 0.028 | 1.659 | 0.01 | ↓ |
| 3-hydroxybutyric acid | 939 | 6.12 | 147 | 1.038 | 0.48 | 1.641 | 0.006 | ↓ |
| uric acid | 896 | 11.71 | 441 | 0.005 | 0.003 | 1.636 | 0.044 | ↓ |
| nicotinamide | 238 | 8.50 | 235 | 0.009 | 0.007 | 1.630 | 0.006 | ↓ |
| heptadecanoic acid | 894 | 11.97 | 117 | 0.022 | 0.009 | 1.618 | 0.011 | ↓ |
| Tagatose 1 | 723 | 10.44 | 307 | 0.003 | 0.001 | 1.596 | 0.007 | ↓ |
| alpha-ketoglutaric acid | 903 | 8.94 | 198 | 0.027 | 0.017 | 1.586 | 0.008 | ↓ |
| Cholestane-3,5,6-triol | 678 | 16.72 | 129 | 0.403 | 0.256 | 1.585 | 0.009 | ↓ |
| piceatannol 2 | 393 | 13.55 | 221 | 0.002 | 0.001 | 1.564 | 0.023 | ↓ |
| N-Acetyl-beta-D-mannosamine 1 | 713 | 11.61 | 202 | 0.002 | 0.001 | 1.561 | 0.013 | ↓ |
| stearic acid | 944 | 12.42 | 117 | 0.767 | 0.520 | 1.547 | 0.007 | ↓ |
| 3-phosphoglycerate | 696 | 10.23 | 357 | 0.008 | 0.002 | 1.530 | 0.017 | ↑ |
| Citramalic acid | 459 | 8.29 | 71 | 0.076 | 0.057 | 1.501 | 0.018 | ↓ |
| fumaric acid | 912 | 7.50 | 245 | 0.024 | 0.010 | 1.483 | 0.010 | ↓ |
| 3-Hydroxypyridine | 645 | 6.05 | 152 | 0.002 | 0.001 | 1.477 | 0.024 | ↓ |
| phenylacetaldehyde 2 | 267 | 6.41 | 89 | 0.002 | 0.001 | 1.467 | 0.013 | ↓ |
| oleic acid | 902 | 12.31 | 339 | 0.085 | 0.052 | 1.447 | 0.013 | ↓ |
| 3-hydroxy-3-methylglutaric acid | 525 | 9.10 | 231 | 0.0024 | 0.0016 | 1.445 | 0.022 | ↓ |
| citric acid | 954 | 10.30 | 273 | 0.538 | 0.466 | 1.415 | 0.016 | ↓ |
| phthalic acid | 412 | 9.72 | 304 | 0.019 | 0.028 | 1.391 | 0.031 | ↑ |
| trans-4-hydroxy-L-proline 2 | 819 | 8.64 | 304 | 0.003 | 0.005 | 1.385 | 0.022 | ↑ |
| allose 1 | 515 | 9.36 | 204 | 0.038 | 0.025 | 1.381 | 0.018 | ↓ |
| Glucose-1-phosphate | 607 | 10.02 | 217 | 0.008 | 0.003 | 1.366 | 0.031 | ↓ |
| 4-hydroxybutyrate | 873 | 6.68 | 233 | 0.0014 | 0.0007 | 1.342 | 0.027 | ↓ |
| Thymol | 377 | 7.27 | 240 | 0.0013 | 0.0005 | 1.338 | 0.037 | ↓ |
| 3-Aminoisobutyric acid 1 | 528 | 8.25 | 174 | 0.014 | 0.011 | 1.338 | 0.037 | ↓ |
| N-Methyl-L-glutamic acid 2 | 383 | 8.92 | 247 | 0.004 | 0.002 | 1.331 | 0.040 | ↓ |
| Threonic acid | 926 | 8.80 | 292 | 0.033 | 0.023 | 1.329 | 0.030 | ↓ |
| oxoproline | 913 | 8.67 | 156 | 1.989 | 2.365 | 1.303 | 0.036 | ↑ |
| p-benzoquinone | 241 | 7.45 | 152 | 0.003 | 0.001 | 1.303 | 0.036 | ↓ |
| lauric acid | 826 | 9.44 | 257 | 0.005 | 0.003 | 1.303 | 0.044 | ↓ |
| fructose 1 | 951 | 10.56 | 103 | 0.27 | 0.194 | 1.089 | 0.049 | ↓ |
| fucose 1 | 763 | 9.79 | 117 | 0.067 | 0.041 | 1.279 | 0.045 | ↓ |
| alpha-ketoisocaproic acid 1 | 732 | 6.51 | 200 | 0.002 | 0.001 | 1.269 | 0.039 | ↓ |
| 3-(2-Hydroxyphenyl)propionic acid | 234 | 9.59 | 221 | 0.003 | 0.002 | 1.248 | 0.043 | ↓ |

(↑↓indicate the variations of differential metabolites of model group compared with normal group)

**Table S2. Differential metabolites between model group and EI30 high-dose group**

| chemical compound | Sim. | R.T.  /min | m/z | Normalized Area | | VIP | P | Trend |
| --- | --- | --- | --- | --- | --- | --- | --- | --- |
|  |  |  |  | MOD | EI30 |  |  |  |
| beta-Mannosylglycerate 2 | 968 | 10.50 | 217 | 0.100 | 0.321 | 2.780 | 0.001 | ↑ |
| Myristic Acid | 939 | 10.52 | 285 | 0.010 | 0.032 | 2.503 | 0.001 | ↑ |
| lauric acid | 826 | 9.44 | 257 | 0.003 | 0.006 | 2.412 | 0.001 | ↑ |
| palmitoleic acid | 913 | 11.42 | 117 | 0.015 | 0.104 | 2.177 | 0.004 | ↑ |
| 1-Monopalmitin | 805 | 13.79 | 371 | 0.002 | 0.003 | 2.007 | 0.005 | ↑ |
| ribose | 896 | 9.49 | 103 | 0.085 | 0.215 | 1.900 | 0.017 | ↑ |
| L-aspartic acid | 933 | 8.59 | 232 | 0.087 | 0.067 | 1.890 | 0.008 | ↓ |
| 4-Hydroxybenzyl cyanide | 242 | 8.74 | 245 | 0.001 | 0.002 | 1.844 | 0.018 | ↑ |
| androsterone 1 | 636 | 13.74 | 91 | 0.052 | 0.105 | 1.839 | 0.012 | ↑ |
| 5,6-dihydrouracil 1 | 567 | 8.46 | 243 | 0.001 | 0.002 | 1.829 | 0.014 | ↑ |
| palmitic acid | 947 | 11.52 | 117 | 0.964 | 1.381 | 1.807 | 0.012 | ↑ |
| pantothenic acid | 714 | 11.22 | 291 | 0.001 | 0.002 | 1.784 | 0.014 | ↑ |
| inosine | 196 | 13.81 | 285 | 0.002 | 0.004 | 1.773 | 0.017 | ↑ |
| leucine | 756 | 6.94 | 158 | 0.396 | 0.489 | 1.755 | 0.017 | ↑ |
| citrulline 1 | 858 | 10.36 | 157 | 0.062 | 0.046 | 1.751 | 0.009 | ↓ |
| 3-hydroxybutyric acid | 939 | 6.12 | 147 | 0.480 | 0.924 | 1.733 | 0.022 | ↑ |
| panthenol 2 | 241 | 11.04 | 71 | 0.013 | 0.029 | 1.724 | 0.019 | ↑ |
| phthalic acid | 412 | 9.72 | 304 | 0.028 | 0.016 | 1.710 | 0.016 | ↓ |
| pentadecanoic acid | 699 | 11.03 | 132 | 0.001 | 0.003 | 1.693 | 0.023 | ↑ |
| Isoleucine | 944 | 7.10 | 158 | 0.113 | 0.208 | 1.676 | 0.027 | ↑ |
| piceatannol 2 | 393 | 13.55 | 221 | 0.0006 | 0.001 | 1.665 | 0.027 | ↑ |
| trans-4-hydroxy-L-proline2 | 819 | 8.64 | 304 | 0.005 | 0.004 | 1.650 | 0.047 | ↓ |
| lysine | 873 | 10.88 | 174 | 0.316 | 0.388 | 1.649 | 0.0235 | ↑ |
| L-cysteine | 829 | 8.83 | 218 | 0.014 | 0.001 | 1.635 | 0.016 | ↓ |
| lactic acid1 | 881 | 5.36 | 51 | 0.001 | 0.020 | 1.614 | 0.042 | ↑ |
| S-carboxymethylcysteine 2 | 338 | 10.43 | 109 | 0.006 | 0.003 | 1.592 | 0.049 | ↓ |
| phenylalanine | 925 | 9.30 | 218 | 0.300 | 0.260 | 1.587 | 0.047 | ↓ |
| phenylacetaldehyde 2 | 267 | 6.41 | 89 | 0.001 | 0.002 | 1.570 | 0.040 | ↑ |
| 4-Hydroxypyridine | 274 | 6.20 | 322 | 0.001 | 0.001 | 1.564 | 0.040 | ↑ |
| D-Altrose 1 | 652 | 10.77 | 273 | 0.029 | 0.038 | 1.536 | 0.046 | ↑ |
| sorbitol | 850 | 10.91 | 333 | 0.006 | 0.010 | 1.487 | 0.041 | ↑ |
| glycerol | 918 | 6.92 | 205 | 0.380 | 0.449 | 1.481 | 0.04 | ↑ |
| kyotorphin 2 | 440 | 13.69 | 151 | 0.0001 | 0.0005 | 1.428 | 0.039 | ↑ |

(↑↓indicate the variations of differential metabolites of EI30 high-dose group compared with model group)

**Ethics Approval (in Chinese)**

**
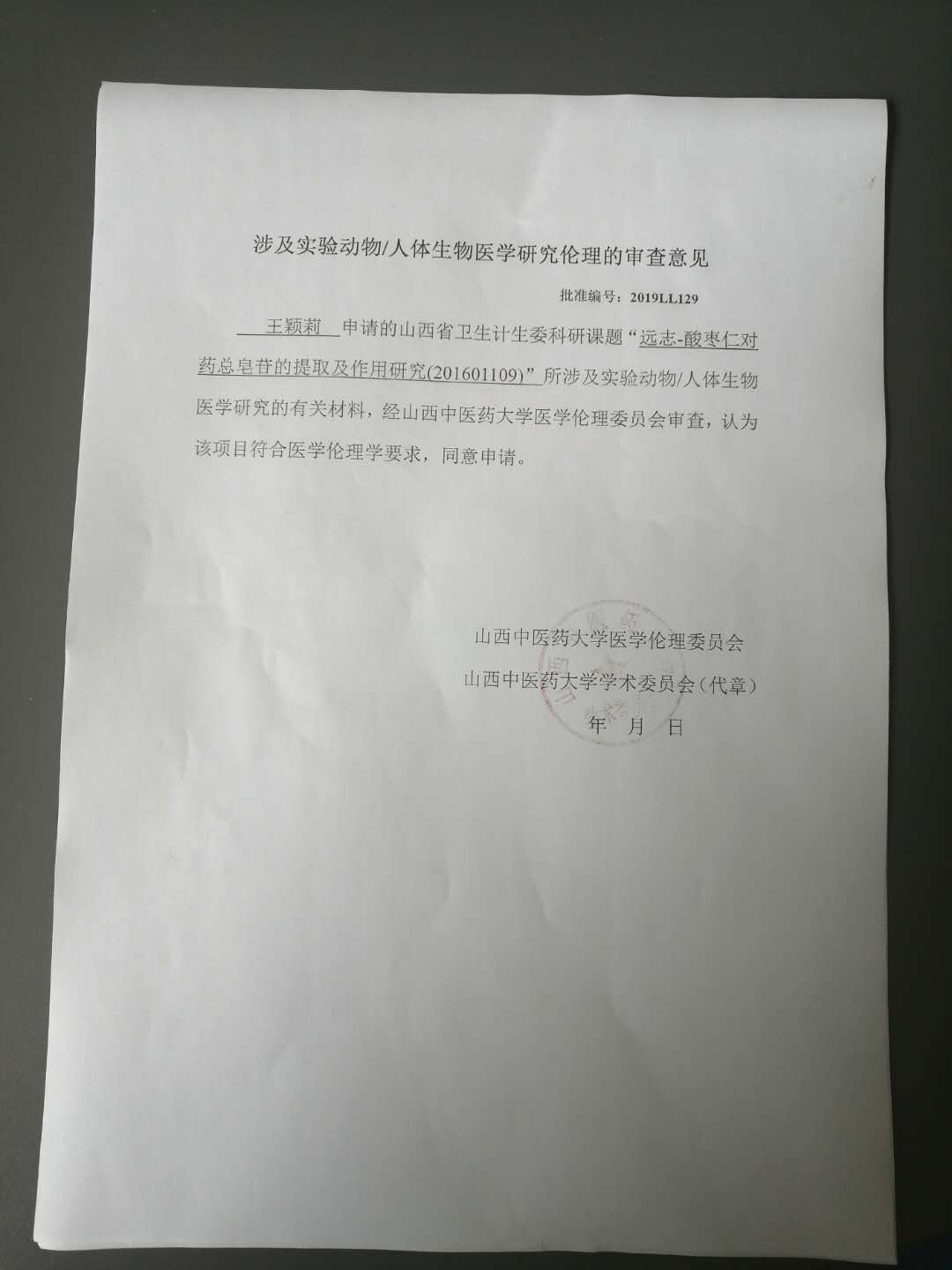
**
